# Supplementary material for: Prognosis of older patients with newly diagnosed AML undergoing antileukemic therapy: A systematic review
Source: PLoS One. 2022 Dec 5;17(12):e0278578. doi: 10.1371/journal.pone.0278578 (PMC9721486; doi:10.1371/journal.pone.0278578)
Supplement: S1 Appendix — (DOCX) [file pone.0278578.s001.docx]

**OVID Medline Epub Ahead of Print, In-Process & Other Non-Indexed Citations, Ovid MEDLINE(R) Daily and Ovid MEDLINE(R) 1946 to Present**

| 1. exp leukemia, myeloid, acute/ |  |
| --- | --- |
| 2. acute myeloid leukemia.mp. |  |
| 3. acute myelogenous leukemia.mp. |  |
| 4. acute nonlymphocytic leukemia.mp. |  |
| 5. exp Leukemia, Myelomonocytic, Acute/ |  |
| 6. or/1-5 |  |
| 7. (acut$ or akut$ or agud$ or aigu$).tw,kf,ot. |  |
| 8. ((promyelocyt$ or promielocitic$ or promyelozyt$ or progranulocyt$) and (leuk?em$ or leuc$)).tw,kf,ot. |  |
| 9. 7 and 8 |  |
| 10. LEUKEMIA, MYELOID/ |  |
| 11. ACUTE DISEASE/ |  |
| 12. 10 and 11 |  |
| 13. (acut$ or akut$ or agud$ or aigu$).tw,kf,ot. |  |
| 14. ((myelo$ or mielo$ or nonlympho$ or granulocytic$) and (leuk?em$ or leuc$)).tw,kf,ot. |  |
| 15. 13 and 14 |  |
| 16. 9 or 12 or 15 |  |
| 17. 6 or 16 |  |
| 18. exp aged/ |  |
| 19. health services for the aged/ or homes for the aged/ or long-term care/ or nursing care/ or exp nursing homes/ |  |
| 20. (advanced years or ageing or aging or elder? or elderly or frail or geriatric? or gerontolog$ or later life or nursing care or nursing home? or old age or oldest old or pensioner? or post-menopausal or postmenopausal or senior or seniors).tw. |  |
| 21. (aged or aging or ageing or elder$ or geriatric$ or gerontolog$).jw,nw. |  |
| 22. ('65 year$' or 'over 65' or 'over 70' or 'over 75' or 'over 80' or 'over 85' or '85 year$').tw. |  |
| 23. or/18-22 |  |
| 24. antileukemic therapy.mp. |  |
| 25. chemotherapy.mp. |  |
| 26. maintenance therapy.mp. |  |
| 27. post?remission therapy.mp. |  |
| 28. transfusion.mp. |  |
| 29. induction.mp. |  |
| 30. consolidation.mp. |  |
| 31. or/24-30 |  |
| 32. incidence.sh. |  |
| 33. exp mortality/ |  |
| 34. follow-up studies.sh. |  |
| 35. prognos:.tw. |  |
| 36. predict:.tw. |  |
| 37. course:.tw. |  |
| 38. or/32-37 |  |
| 39. 17 and 23 and 31 and 38 |  |
| 40. limit 39 to yr="2000 -Current" |  |

**Embase**1974 to 2020 July 15

| 1. exp acute myeloid leukemia/ |  |
| --- | --- |
| 2. acute myeloid leukemia.mp. |  |
| 3. acute myelogenous leukemia.mp. |  |
| 4. acute nonlymphocytic leukemia.mp. |  |
| 5. or/1-4 |  |
| 6. (acut$ or akut$ or agud$ or aigu$).tw,kw,hw,ot. |  |
| 7. ((promyelocyt$ or promielocitic$ or promyelozyt$ or progranulocyt$) and (leuk?em$ or leuc$)).tw,kw,hw,ot. |  |
| 8. 6 and 7 |  |
| 9. myeloid leukemia/ |  |
| 10. acute disease/ |  |
| 11. 9 and 10 |  |
| 12. (acut$ or akut$ or agud$ or aigu$).tw,kw,hw,ot. |  |
| 13. ((myelo$ or mielo$ or nonlympho$ or granulocytic$) and (leuk?em$ or leuc$)).tw,kw,hw,ot. |  |
| 14. 12 and 13 |  |
| 15. 8 or 11 or 14 |  |
| 16. 5 or 15 |  |
| 17. exp aged/ |  |
| 18. exp elderly care/ |  |
| 19. (advanced years or ageing or aging or elder? or elderly or frail or geriatric? or gerontolog$ or later life or nursing care or nursing home? or old age or oldest old or pensioner? or post-menopausal or postmenopausal or senior or seniors).tw. |  |
| 20. (old$ adj3 (adult? or female? or male? or men or people or person or women)).tw. |  |
| 21. ('65 year$' or 'over 55' or 'over 65' or 'over 70' or 'over 75' or 'over 80' or 'over 85' or '85 year$').tw. |  |
| 22. (aged or aging or ageing or elder$ or geriatric$ or gerontolog$).jw. |  |
| 23. or/17-22 |  |
| 24. follow-up.mp. |  |
| 25. prognos:.tw. |  |
| 26. ep.fs. |  |
| 27. or/24-26 |  |
| 28. antileukemic therapy.mp. |  |
| 29. chemotherapy.mp. |  |
| 30. maintenance therapy.mp. |  |
| 31. post?remission therapy.mp. |  |
| 32. induction.mp. |  |
| 33. consolidation.mp. |  |
| 34. transfusion.mp. |  |
| 35. or/28-34 |  |
| 36. 16 and 23 and 27 and 35 |  |
| 37. remove duplicates from 36 |  |
| 38. limit 37 to yr="2000 -Current" |  |
