# Supplementary material for: Prognosis of older patients with newly diagnosed AML undergoing antileukemic therapy: A systematic review
Source: PLoS One. 2022 Dec 5;17(12):e0278578. doi: 10.1371/journal.pone.0278578 (PMC9721486; doi:10.1371/journal.pone.0278578)
Supplement: S2 Appendix — (DOCX) [file pone.0278578.s002.docx]

| **ID** | **Author** | **Year** | **Source** | **Country/ies(Trials)** | **Recruitment** | **Follow-up (years, median/max)** | **Sample size in the model** | **Age (years)** | **Female (%)** | **Predictors** | **Outcomes** |
| --- | --- | --- | --- | --- | --- | --- | --- | --- | --- | --- | --- |
| 1 | Delia | 2015 | Multicentre | Italy | 2012 | 3.33 | 90 | 75 (65 to 89) | 47 | Performance status | Long-term mortality |
| 2 | Gbadamosi | 2018 | Single centre | USA | 2010-2015 | 6.67 | 137 | 65 (14.7) | 43.1 | Age | Long-term mortality |
| 3 | Collinge | 2018 | Single centre | France | 1986-2014 | 15 | 169 | 75 (70 to 93) | 43.8 | Age | Long-term mortality |
| 5 | Dalley | 2001 | Single centre | UK | 1969-1999 | 11 | 288 | 66 (60 to 83) | 41.3 | Age | Long-term mortality |
| 9 | Xu | 2014 | Single centre | China | 2006-2013 | 7.08 | 152 | 68 (60 to 94) | 40.1 | Age | Long-term mortality |
| 12 | Zhang | 2017 | Single centre | China | 2000-2014 | 4.11 | 74 | 68 (60 to 82) | 36.2 | Performance status, Comorbidity | Long-term mortality |
| 20 | Tawfik | 2016 | Single centre | USA | 2002-2009 | 7 | 144 | 70.3 (6.6) | 46.5 | Comorbidity | Long-term mortality and short-term mortality |
| 25 | Oh | 2017 | Multicentre | Korea | 2010-2015 | 3.33 | 86 | 73 (65 to 86) | 51.2 | Age, Performance status, Comorbidity | Long-term mortality |
| 35 | Wang | 2016 | Single centre | China | 2009-2015 | 1.08 | 103 | 63 (60 to 78) | 46.6 | Age | Long-term mortality |
| 40 | Shacham-Abulafia | 2016 | Single centre | Israel | 2007-2014 | 6.67 | 62 | 67 (60 to 79) | 35 | Age, Performance status | Long-term mortality |
| 41 | Kahl | 2016 | Multicentre | Germany | 1998-2005 | 10 | 410 | 66 (60 to 69) | 48 | Performance status | Long-term mortality |
| 47 | Alibhai | 2015 | Multicentre | Canada | 2008-2012 | 1 | 97 | 60 to 81 | 34 | Age, performance status, comorbidity | Quality of life measured by QLQ-30 |
| 48 | Fattoum | 2015 | Single centre | France | 1985-1999 and 2000 to 2014 | 5 | 682 (499 and 183) | 73 (60 to 86) | 47.5 | Age, Performance status | Long-term mortality |
| 50 | Medeiros | 2015 | Multicentre | USA | 2000-2009 | 11 | 3327 | 75 | 44.9 | Age, Performance status, Comorbidity | Long-term mortality |
| 53 | Ramos | 2015 | Multicentre | Spain | 2006-2010 | 4 | 371 (110 and 261) | 75 (31 to 93) | 39 | Age, Performance status | Long-term mortality |
| 57 | Hulegardh | 2015 | Multicentre | Sweden | 1997-2006 | 9.6 | 3363 | 70 (56 to 79) | 50 | Age | Long-term mortality |
| 58 | Budziszewska | 2015 | Multicentre | Poland | 2005-2009 | 0.94 | 509 | 69 (61 to 93) | 44 | Age | Short-term mortality (8-week mortality) |
| 62 | Ostgard | 2015 | Multicentre | Denmark | 2000-2012 | 1.33 | 783 | >=60 | NR | Performance status, Comorbidity | Long-term mortality and short-term mortality |
| 65 | Bories | 2014 | Multicentre | France | 2007-2010 | 2.92 | 210 (95 and 115) | 72 (63 to 81) | 37 | Age, | Long-term mortality |
| 76 | Thepot | 2014 | Multicentre | France | 2004-2019 | 2.63 | 149 | 74 (31 to 91) | 40.9 | Performance status | Long-term mortality |
| 86 | van der Helm | 2013 | Multicentre | Netherlands | 2010-2012 | 2 | 55 | 73 (59 to 84) | 25 | Performance status | Long-term mortality |
| 88 | Brunner | 2013 | Single centre | USA | 1992-2011 | 10.96 | 97 | 67 (60-87) | 48.5 | Age | Long-term mortality |
| 89 | Shi | 2013 | Single centre | China | 2000-2010 | 2.19 | 116 | 69 (60-87) | 41.4 | Age, Performance status, Comorbidity | Long-term mortality |
| 90 | Zhao | 2013 | Single centre | China | 2007-2011 | 4 | 211 | 63 (55-80) | 37.9 | Age, Performance status | Long-term mortality |
| 92 | Klepin | 2013 | Single centre | USA | 2009-2011 | 1.64 | 74 | 70 (6.2) | 45.9 | Age, Performance status, Comorbidity | Long-term mortality |
| 93 | Okuyama | 2013 | Multicentre | Japan, hungary, Netherlands, Austria | 2000-2011 | 4.11 | 69 | >=60 | NR | Performance status, Comorbidity | Long-term mortality |
| 97 | Gardin | 2013 | Multicentre | ALFA-9801 and ALFA-9803 | 1999-2006 | 7.5 | 727 | 67 (50 to 85) | 45.7 | Age, Performance status (ECOG: 2+ VS <2) | Long-term mortality |
| 99 | Kim | 2013 | Multicentre | Korea | 2007-2009 | 1.24 | 108 | 68.4 (60.3 to 81.2) | 40.7 | Age, Performance status, Comorbidity | Long-term mortality and short-term mortality |
| 105 | Djunic | 2012 | Single centre | Serbia | NR | 4.8 | 81 | >=55 | 52 | Comorbidity | Long-term mortality and short-term mortality |
| 106 | Colovic | 2012 | Single centre | Serbia | 2001-2006 | 4 | 87 | 68 (65 to 77) | 32.2 | Performance status | Long-term mortality |
| 122 | Baer | 2011 | Multicentre | CALGB 9720 | 1998-2002 | 10 | 610 | 71 (60 to 90) | 40 | Age, Performance status | Long-term mortality |
| 127 | Krug | 2010 | Multicentre | AMLCG1999 | 1999-2009 | 0.17 | 1406 | 71 (60-90) | NR | Age | Short-term mortality (60-day mortality) |
| 132 | Pigneux | 2010 | Multicentre | BGMT-95+GOELAMS-SA4+LAMSA-2002 | 1995-2005 | 4 | 847 | 69 (60 to 86) | 49 | Age, Performance status | Long-term mortality |
| 133 | Rollig | 2010 | Multicentre | AML96 | 1996-2004 | 5.67 | 909 | 67 (61 to 87) | 48.4 | Performance status | Long-term mortality |
| 140 | Prebet | 2009 | Multicentre | France | NR | 4 | 147 | 67 (60 to 82) | 51.7 | Performance status | Long-term mortality |
| 144 | Wheatley | 2009 | Multicentre | AML 14 | 1990-2006 | 3 | 2208 (1071 and 1137) | NR | NR | Age, Performance status | Long-term mortality |
| 147 | Burnett | 2009 | Multicentre | UK | 1998-2006 | 4.75 | 1273 | 67 (44 to 88) | 39.4 | Age | Long-term mortality |
| 154 | Sekeres | 2009 | Multicentre | USA | 1994-2005 | 4 | 664 | 69.1 (56.0) | NR | Age, Performance status | Long-term mortality |
| 156 | Malfuson | 2008 | Multicentre | ALFA-9803 | 1999-2006 | 2.83 | 416 | 72 (65-85) | 45.9 | Age (75+ VS <75 years), Performance status (2+ VS <2), Comorbidity (HCT-CI: 3+ VS <3) | Long-term mortality |
| 159 | Tsimberidou | 2008 | Single centre | USA | 1990-2005 | 2.94 or 3.27 | 703 | >=60 | 37.3 | Age, Performance status | Long-term mortality |
| 166 | Baz | 2007 | Single centre | USA | 1997-2005 | 4.81 | 182 | 71. 5 (60-92) | 40 | Age | Long-term mortality |
| 171 | Lancet | 2007 | Multicentre | USA | 2001-2005 | 2.74 | 158 | 74 (34 to 85) | 40 | Age, Performance status | Long-term mortality |
| 177 | Frohling | 2006 | Multicentre | Germany and Austrian/ AML HD98-B trial | NR | 4.75 | 361 | 67 (61 to 84) | 46 | Age | Long-term mortality |
| 185 | Farag | 2006 | Multicentre | USA | 1984-1999 | 10.9 | 635 | 68 (60 to 86) | 45.5 | Age | Long-term mortality |
| 186 | Kantarjian | 2006 | Single centre | USA | 1980-2004 | 8 | 998 | 71 (65 to 89) | NR | Age, Performance status | Long-term mortality and short-term mortality |
| 193 | Gupta | 2005 | Single centre | Canada | 1998-2002 | 2.75 | 117 | 67 (60-82) | 42.7 | Age, Performance status | Long-term mortality |
| 198 | Schlenk | 2004 | Multicentre | Germany | 1998-2001 | 2.83 | 242 | 66 (61 to 84.5) | 47.1 | Age | Long-term mortality |
| 199 | Schoch | 2004 | Multicentre | AMLCG 1992, AMLCG 1999 and AMLCG APL trials | NR | 1.31 | 537 | >=60 | NR | Age | Long-term mortality |
| 200 | Vey | 2004 | Single centre | France | 1990-2001 | 2.33 | 310 | 72 (65 to 91) | 46.5 | Age | Long-term mortality |
| 204 | Pulsoni | 2004 | Multicentre | Italy | 1992-1998 | 10.42 | 1005 | 69 | NR | Age, Performance status | Long-term mortality |
| 216 | Anderson | 2002 | Multicentre | SWOG-9333 | 1995-1998 | 5 | 328 | 67.5 (56 to 86) | 43.9 | Age, Performance status | Long-term mortality |
| 222 | Wahlin | 2001 | Single centre | Sweden | 1982-1998 | 10 | 211 | 72.8 (60 to 90) | 44.1 | Age | Long-term mortality |
| 224 | Goldstone | 2001 | Multicentre | AML 11 | 1990-1998 | 5 | 1209 | 66 (44-91) | 44 | Age, Performance status | Long-term mortality and short-term mortality |
| 227 | Yoshida | 2001 | Multicentre | Japan | 1987-1994 | 10 | 112 | 72 (60 to 92) | 40.2 | Performance status | Long-term mortality |
| 232 | Astrom | 2000 | Multicentre | Sweden | 1987-1992 | 11.67 | 214 | 69.5 | NR | Age | Long-term mortality |
| 242 | Gangatharan | 2013 | Multicentre | Australia | 1991-2005 | 9.15 | 898 | 67 (17 to 94) | 43.5 | Age | Long-term mortality |
| 260 | Sherman | 2013 | Single centre | USA | 2006-2011 | 5 | 101 | 72 | 37.6 | Performance status, Comorbidity | Long-term mortality |
| 281 | Chen | 2016 | Single centre | China | 2003-2015 | 2.26 | 248 | 66 (60 to 87) | 44.8 | Age, Performance status | Long-term mortality |
| 282 | Dombret | 2015 | Multicentre | Europe/Israel, etc. | 2010-2014 | 2.03 | 488 | 75 (64 to 91) | 41 | Age, Performance status | Long-term mortality |
| 283 | Takahashi | 2016 | Single centre | USA | 2002-2012 | 11.67 | 190 | 67.5 (60 to 85) | 34.2 | Age, Performance status | Long-term mortality |
| 284 | Boddu | 2017 | Single centre | USA | 1990-2015 | 20 | 931 | 68 (60 to 75) | NR | Age | Long-term mortality |
| 319 | Oran | 2012 | Multicentre | United States | 2000-2007 | 5 | 2113 | >=65 | 44 | Age, Comorbidity | Short-term mortality |
| 321 | Gardin | 2007 | Multicentre | ALFA-9803 | 1999-2006 | 2.83 | 416 | 72 (65-85) | 45.9 | Age (5-year increment), Performance status (2+ VS <2) | Long-term mortality, short-term mortality |
| 326 | Pautas | 2010 | Multicentre | ALFA-9801 | 1999-2006 | 4.1 | 468 | 60 (50-70) | 44.7 | Age (60+ VS <60years), Performance status (2+ VS <2) | Long-term mortality, |
| 328 | Tassara | 2014 | Multicentre | Germany and Austria | 2004-2006 | 7.03 | 186 | 68.2(61 to 83.7) | 47.3 | Age | Long-term mortality |
| 331 | Cripe | 2010 | Multicentre | Eastern Cooperative Oncology Group 3999 | 2002-2005 | 4.18 | 433 | 69 (65 to 73) | 43.3 | Performance status | Long-term mortality |
| 348 | Timilshina | 2016 | Multicentre | Canada | 2008-2012 | 1 | 97 | 68 (>=60) | 34.1 | Age, Performance status, Comorbidity | Long-term mortality |
| 351 | Ostronoff | 2015 | Multicentre | SWO Gtrials (S9333, S9031, S9500, and S0106) | 1992-2009 | 14 | 156 | 60 (55 to 83) | 47.4 | Performance status | Long-term mortality |
| 356 | Amadori | 2013 | Multicentre | AML-17 | 2002-2007 | 5.2 | 472 | 67 (61 to 75) | 43.4 | Age, Performance status | Long-term mortality |
| 1216 | Falantes | 2017 | Multicentre | European | 2011-2014 | 2.78 | 710 | 75 (60–93) | 36.9 | Age, Performance status | Long-term mortality |
| 1217 | Guo | 2018 | Single centre | China | 2014-2017 | 1.25 | 121 | 66.8 (4.1) | 44.6 | Age | Long-term mortality |
| 1218 | Heiblig | 2019 | Single centre | France | 2000-2016 | 1.67 | 495 | 69 (60-93) | 43 | Age, Performance status | Long-term mortality |
| 1222 | Prassek | 2018 | Multicentre | AMLCG-1999 trial | 1999-2011 | 7 | 151 | 76 (75-86) | 46 | Performance status | Long-term mortality |
| 1224 | Wan | 2019 | Single centre | China | 2007-2017 | 11 | 68 | 70 (60-91) | 34.5 | Age, comorbidities (p value), frail (p value) | Long-term mortality |
| 1226 | Bocchia | 2019 | Multicentre | Italy | since 2013 | 1.03 | 306 | 75 (65-90) | NR | Age | Long-term mortality |
| 1227 | Fili | 2019 | Multicentre | Italy | 2015-2017 | 1.67 | 75 | 74 (65-84) | 46.7 | Age, Performance status | Long-term mortality |
| 1228 | Huang | 2018 | Single centre | China | 2011-2016 | 5 | 117 | 67 (60-87) | 45.3 | Age, Performance status | Long-term mortality |
| 1229 | Vachhani | 2018 | Single centre | United States | 2008-2016 | 8.5 | 201 | 71 (60-93) | 33.3 | Age, Performance status, comorbidities | Long-term mortality |
| 1231 | Chen | 2018 | Single centre | China | 2012-2017 | 5 | 70 | 71 (60-81) | 41.4 | Age, Performance status | Long-term mortality |
| 2572 | Osterroos | 2020 | Single centre | Swedish | 2008-2018 | 3 | 1529 | 70 (60 - 87) | 44.7 | Age, Performance status | Long-term mortality |
| 2068 | Hu | 2020 | Single centre | China | 2014-2016 | 2 | 68 | 70 (60 - 89) | 45.6 | Age | Long-term mortality |
| 2142 | Wei | 2020 | Multicentre | America et etc. | 2017-2018 | 1 | 210 | 76 (36-93) | 46.9 | Age, Performance status | Long-term mortality |
| 2290 | Zeidan | 2020 | Single centre | USA | 2001-2013 | 0.64 | 2263 | 77 (72-82) | 41.4 | Age, comorbidities | Long-term mortality |
| 2349 | Hong | 2020 | Single centre | China | 2011-2018 | 1 | 125 | 66 (60-86) | 46.4 | Age | Long-term mortality |
| 2658 | Apel | 2021 | Multicentre | Isreal | 2016-2020 | 0.73 | 133 | 77 (52-95) | 47 | Performance status | Long-term mortality |
| 2631 | Budziszewska | 2021 | Multicentre | Poland | 2017-2019 | 3.3 | 117 | 70 (60-87) | 56 | Age, Performance status, comorbidities | Long-term mortality |
| 2632 | Heiblig | 2021 | Multicentre | France | 2012-2016 | 1.67 | 138 | 66 (60–78) | NR | Age | Long-term mortality |
| 2620 | Peipert | 2021 | Multicentre | USA etc. | 2015-2019 | 1.64 | 326 | 75 (51-92) | 47 | Age | Long-term mortality |
| 2621 | Zhang | 2021 | Multicentre | China | 2010-2019 | 0.73 | 228 | 71 (60–91) | 37.3 | Performance status | Long-term mortality |
| 2191 | Pepe | 2020 | Single centre | Italy | 2007-2019 | 0.6 | 110 | 75 (58-87) | 32.7 | Age, comorbidities | Long-term mortality |
