# Supplementary material for: Prognosis of older patients with newly diagnosed AML undergoing antileukemic therapy: A systematic review
Source: PLoS One. 2022 Dec 5;17(12):e0278578. doi: 10.1371/journal.pone.0278578 (PMC9721486; doi:10.1371/journal.pone.0278578)
Supplement: S3 Appendix — (DOCX) [file pone.0278578.s003.docx]

| **ID** | **First Author** | **Year** | **Predictors and outcomes** | **Study Participation** | **Study Attrition** | **Prognostic Factor Measurement** | **Outcome Measurement** | **Study Confounding** | **Statistical Analysis and Reporting** | **Overall quality** |
| --- | --- | --- | --- | --- | --- | --- | --- | --- | --- | --- |
| 1 | Delia | 2015 | All reported | moderate | low | Low | low | moderate | low | moderate |
| 2 | Gbadamosi | 2018 | All reported | moderate | low | Low | low | moderate | low | moderate |
| 3 | Collinge | 2018 | All reported | moderate | low | Low | low | moderate | high | moderate |
| 5 | Dalley | 2001 | All reported | moderate | low | Low | low | moderate | high | moderate |
| 9 | Xu | 2014 | All reported | moderate | low | Low | low | moderate | high | moderate |
| 12 | Zhang | 2017 | All reported | high | low | Low | low | moderate | moderate | moderate |
| 20 | Tawfik | 2016 | All reported | moderate | low | Low | low | low | low | low |
| 25 | Oh | 2017 | All reported | moderate | low | Low | low | high | low | moderate |
| 35 | Wang | 2016 | All reported | moderate | low | Low | low | moderate | low | moderate |
| 40 | Shacham-Abulafia | 2016 | All reported | high | low | Low | low | high | low | high |
| 41 | Kahl | 2016 | All reported | moderate | low | Low | low | moderate | high | moderate |
| 47 | Alibhai | 2015 | All reported | moderate | low | Low | low | moderate | high | moderate |
| 48 | Fattoum | 2015 | Age and long-term mortality in 1985 to 1999 cohort | moderate | low | moderate | low | moderate | high | moderate |
| 48 | Fattoum | 2015 | Age and long-term mortality in 2000 to 2014 cohort | moderate | low | Low | low | moderate | high | moderate |
| 48 | Fattoum | 2015 | Ps and long-term mortality in 2000 to 2014 cohort | moderate | low | Low | low | low | low | low |
| 50 | Medeiros | 2015 | All reported | moderate | low | Low | low | low | moderate | moderate |
| 53 | Ramos | 2015 | PS and long term mortality in training cohort | moderate | low | Low | low | moderate | high | moderate |
| 53 | Ramos | 2015 | Age in validation cohort | high | low | Low | low | moderate | high | high |
| 53 | Ramos | 2015 | PS in validation cohort | high | low | Low | low | low | high | high |
| 57 | Hulegardh | 2015 | All reported | high | low | Low | low | moderate | moderate | moderate |
| 58 | Budziszewska | 2015 | All reported | high | moderate | moderate | low | moderate | moderate | moderate |
| 62 | Ostgard | 2015 | All reported | moderate | low | Low | low | moderate | moderate | moderate |
| 65 | Bories | 2014 | Age and long-term mortality in azacitidine arm | moderate | low | Low | low | moderate | low | moderate |
| 65 | Bories | 2014 | Age and long-term mortality in intensive chemotherapy arm | moderate | low | Low | low | moderate | low | moderate |
| 76 | Thepot | 2014 | All reported | high | low | Low | low | low | low | low |
| 86 | van der Helm | 2013 | All reported | moderate | low | Low | low | moderate | low | moderate |
| 88 | Brunner | 2013 | All reported | moderate | low | Low | low | moderate | low | moderate |
| 89 | Shi | 2013 | All reported | moderate | low | Low | low | moderate | high | moderate |
| 90 | Zhao | 2013 | Age and long-term mortality | high | low | Low | low | high | high | high |
| 90 | Zhao | 2013 | PS and long term mortality | high | low | Low | low | moderate | high | high |
| 92 | Klepin | 2013 | All reported | moderate | low | Low | low | low | low | low |
| 93 | Okuyama | 2013 | All reported | moderate | low | Low | low | moderate | high | moderate |
| 97 | Gardin | 2013 | All reported | moderate | low | Low | low | low | high | moderate |
| 99 | Kim | 2013 | All reported | low | moderate | Low | low | moderate | low | moderate |
| 105 | Djunic | 2012 | All reported | moderate | low | Low | low | moderate | low | moderate |
| 106 | Colovic | 2012 | All reported | moderate | low | Low | low | moderate | high | moderate |
| 122 | Baer | 2011 | All reported | low | low | Low | low | low | low | low |
| 127 | Krug | 2010 | All reported | low | low | low | low | moderate | high | moderate |
| 132 | Pigneux | 2010 | All reported | moderate | low | Low | low | low | low | low |
| 133 | Rollig | 2010 | All reported | low | low | Low | low | moderate | low | low |
| 140 | Prebet | 2009 | All reported | moderate | low | Low | low | high | low | moderate |
| 144 | Wheatley | 2009 | All reported | low | low | Low | low | low | high | low |
| 147 | Burnett | 2009 | All reported | low | low | Low | low | moderate | high | moderate |
| 154 | Sekeres | 2009 | All reported | moderate | low | Low | low | low | high | moderate |
| 156 | Malfuson | 2008 | All reported | low | low | Low | low | low | low | low |
| 159 | Tsimberidou | 2008 | All reported | moderate | low | Low | low | moderate | low | moderate |
| 166 | Baz | 2007 | All reported | moderate | low | Low | low | moderate | low | moderate |
| 171 | Lancet | 2007 | All reported | moderate | low | Low | low | low | high | moderate |
| 177 | Frohling | 2006 | All reported | moderate | low | Low | low | moderate | low | moderate |
| 185 | Farag | 2006 | All reported | moderate | low | Low | low | moderate | low | moderate |
| 186 | Kantarjian | 2006 | All reported | moderate | low | Low | low | low | high | moderate |
| 193 | Gupta | 2005 | All reported | moderate | low | Low | low | low | low | low |
| 198 | Schlenk | 2004 | All reported | low | low | Low | low | moderate | low | low |
| 199 | Schoch | 2004 | All reported | moderate | low | Low | low | moderate | high | moderate |
| 200 | Vey | 2004 | All reported | moderate | low | Low | low | high | low | moderate |
| 204 | Pulsoni | 2004 | All reported | moderate | low | Low | low | moderate | low | moderate |
| 216 | Anderson | 2002 | All reported | low | low | Low | low | low | high | low |
| 222 | Wahlin | 2001 | All reported | moderate | low | Low | low | moderate | low | moderate |
| 224 | Goldstone | 2001 | All reported | moderate | low | Low | low | low | high | moderate |
| 227 | Yoshida | 2001 | All reported | moderate | low | Low | low | high | high | high |
| 232 | Astrom | 2000 | All reported | moderate | low | Low | low | high | high | high |
| 242 | Gangatharan | 2013 | All reported | moderate | low | Low | low | high | high | high |
| 260 | Sherman | 2013 | All reported | moderate | low | Low | low | low | low | low |
| 281 | Chen | 2016 | All reported | moderate | low | Low | low | low | low | low |
| 282 | Dombret | 2015 | All reported | low | moderate | Low | low | low | moderate | moderate |
| 283 | Takahashi | 2016 | All reported | moderate | low | Low | low | low | low | low |
| 284 | Boddu | 2017 | All reported | moderate | low | Low | low | moderate | low | moderate |
| 319 | Oran | 2012 | All reported | moderate | low | Low | low | high | low | moderate |
| 321 | Gardin | 2007 | All reported | low | low | Low | low | moderate | low | low |
| 326 | Pautas | 2010 | All reported | moderate | low | Low | low | low | high | moderate |
| 328 | Tassara | 2014 | All reported | low | low | Low | low | moderate | high | moderate |
| 331 | Cripe | 2010 | All reported | moderate | low | Low | low | moderate | low | moderate |
| 348 | Timilshina | 2016 | All reported | moderate | low | Low | low | high | low | moderate |
| 351 | Ostronoff | 2015 | All reported | moderate | low | Low | low | moderate | low | moderate |
| 356 | Amadori | 2013 | All reported | low | low | low | low | moderate | low | low |
| 1216 | Falantes | 2017 | All reported | Moderate | Low | Low | Low | Low | Low | Low |
| 1217 | Guo | 2018 | All reported | Low | moderate | Low | Low | Moderate | Low | moderate |
| 1218 | Heiblig | 2019 | Age and long-term mortality | Moderate | Low | Low | Low | Low | Moderate | Moderate |
| 1218 | Heiblig | 2019 | PS and long -term mortality | Moderate | Low | Moderate | Low | Low | Moderate | Moderate |
| 1222 | Prassek | 2018 | All reported | Low | Low | Low | Low | moderate | Low | Low |
| 1224 | Wan | 2019 | Age and long-term mortality | High | High | Low | Low | High | Moderate | High |
| 1226 | Bocchia | 2019 | All reported | Moderate | Low | Low | Low | Moderate | Moderate | Moderate |
| 1227 | Fili | 2019 | All reported | Moderate | Low | Low | Low | Moderate | High | Moderate |
| 1228 | Huang | 2018 | Age and long-term mortality | Low | Moderate | Low | Low | High | High | High |
| 1228 | Huang | 2018 | PS and long-term mortality | Low | Moderate | Low | Low | Moderate | Low | Moderate |
| 1229 | Vachhani | 2018 | All reported | Moderate | Low | Low | Low | Low | Low | Low |
| 1231 | Chen | 2018 | All reported | High | Moderate | Low | Low | Low | High | High |
| 2572 | Osterroos | 2020 | All reported | moderate | low | low | low | moderate | low | moderate |
| 2068 | Hu | 2020 | All reported | moderate | low | low | low | moderate | moderate | moderate |
| 2142 | Wei | 2020 | All reported | low | low | low | low | moderate | low | low |
| 2290 | Zeidan | 2020 | All reported | moderate | low | low | low | moderate | low | moderate |
| 2349 | Hong | 2020 | All reported | moderate | low | low | low | moderate | moderate | moderate |
| 2658 | Apel | 2021 | All reported | moderate | low | low | low | moderate | moderate | moderate |
| 2631 | Budziszewska | 2021 | Age and comorbidity | low | low | low | low | low | moderate | low |
| 2631 | Budziszewska | 2021 | PS and long-term mortality | low | low | low | low | high | moderate | moderate |
| 2632 | Heiblig | 2021 | All reported | moderate | low | low | low | high | moderate | moderate |
| 2620 | Peipert | 2021 | All reported | moderate | low | low | low | moderate | moderate | moderate |
| 2621 | Zhang | 2021 | All reported | moderate | low | low | low | low | moderate | moderate |
| 2191 | Pepe | 2020 | All reported | moderate | low | low | low | high | high | high |
