# Supplementary material for: Prognosis of older patients with newly diagnosed AML undergoing antileukemic therapy: A systematic review
Source: PLoS One. 2022 Dec 5;17(12):e0278578. doi: 10.1371/journal.pone.0278578 (PMC9721486; doi:10.1371/journal.pone.0278578)

Age and long-term mortality-HR

Figure 1：Age predicts long-term mortality among older patients with AML
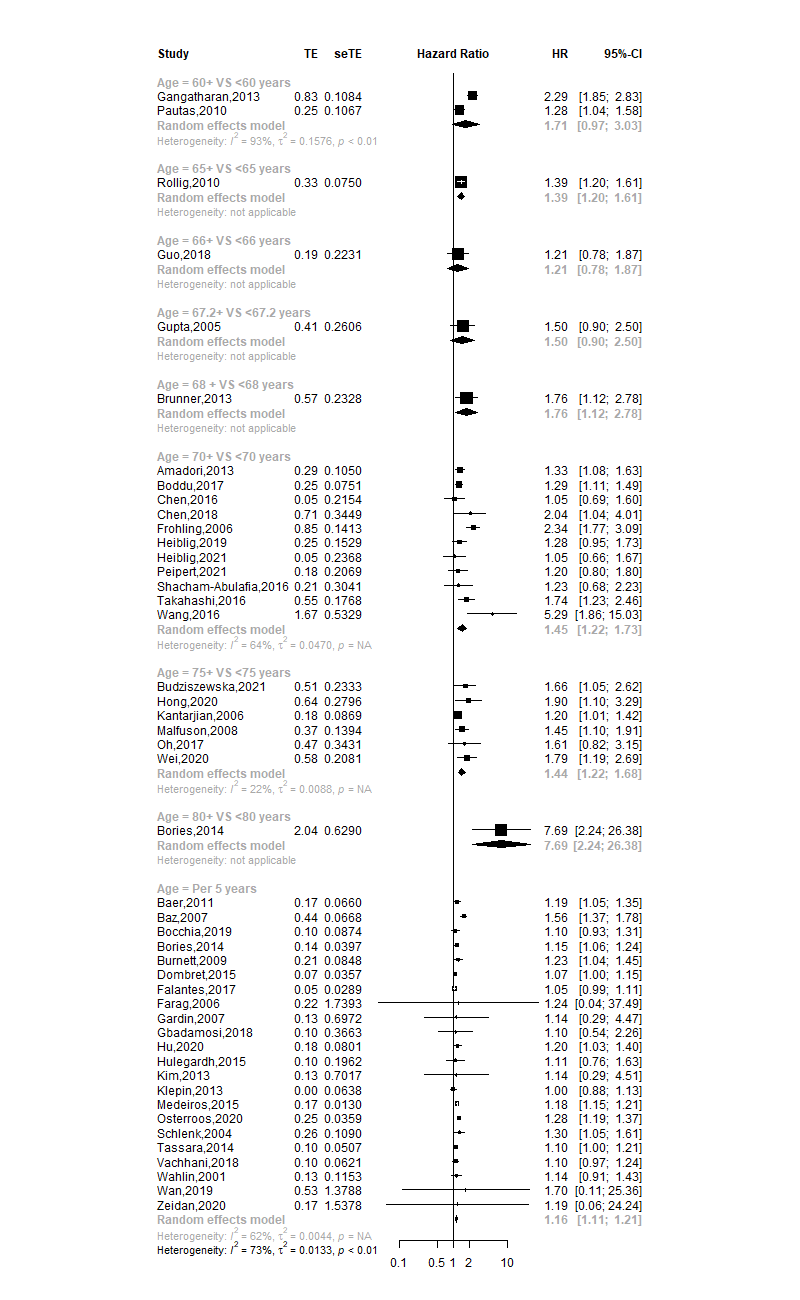


Age and long-term mortality-OR and RR

Figure 1: Age and long-term mortality among older patients with AML-OR


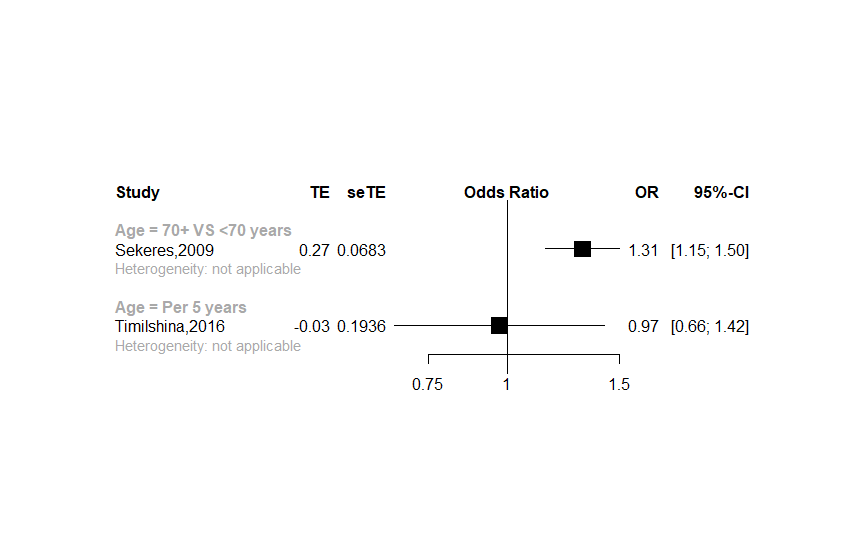


Figure 2: Age and long-term mortality among older patients with AML-RR


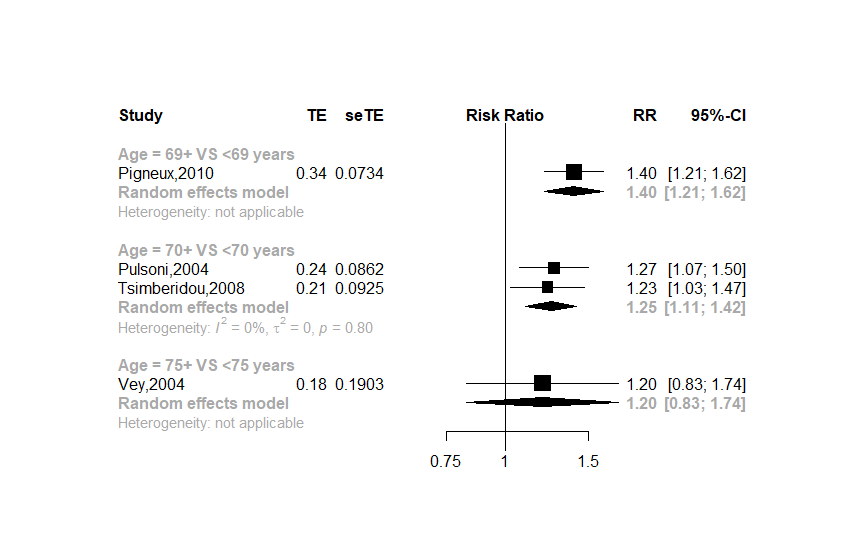


Age and OS-All study all reported relative effect (HR, OR, RR)

Figure 1：Age and long-term mortality among older patients with AML-All (HR and OR)


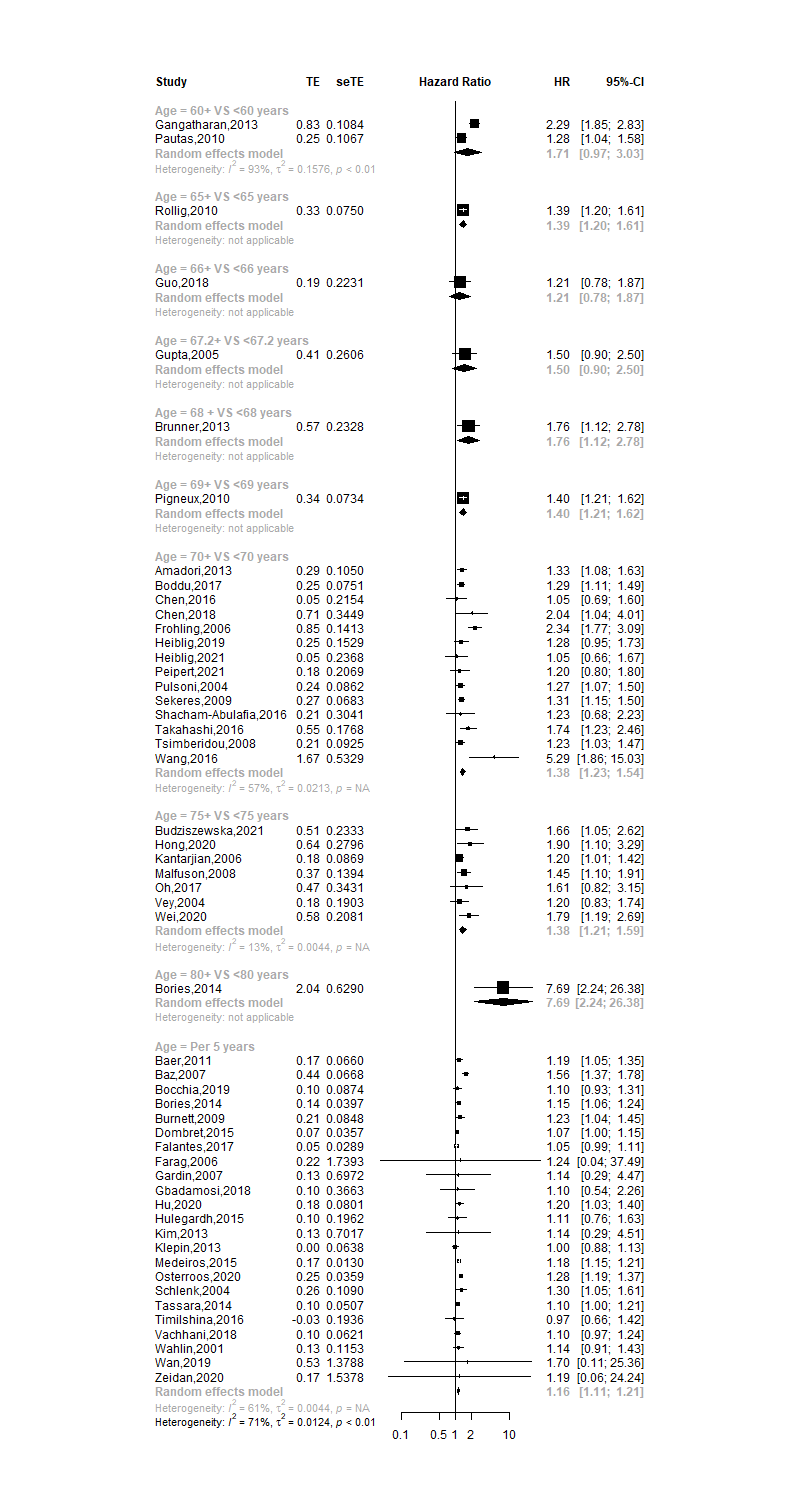


Age and short-term mortality

**Figure 1：Age and short-term mortality (treatment or induction-mortality) among older patients with AML-HR**


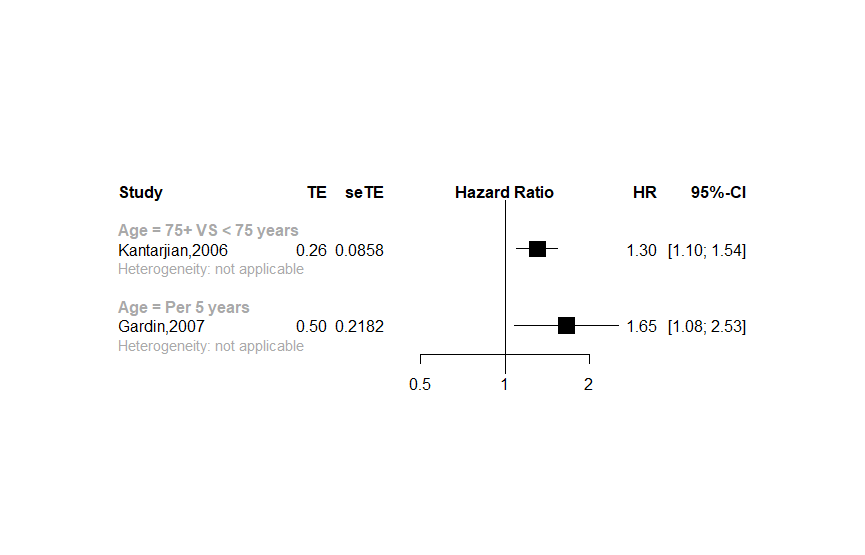


**Figure 2：Age and short-term mortality (treatment or induction-mortality) among older patients with AML-RR**


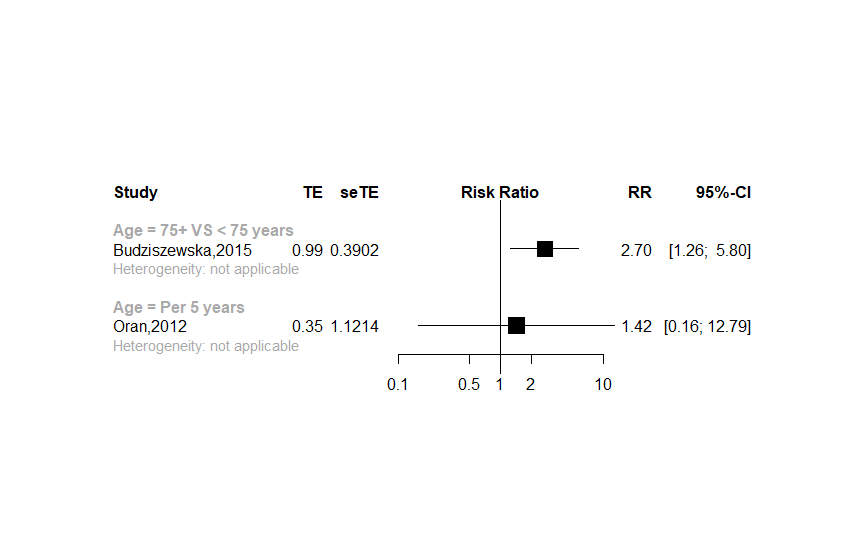


**Figure 3：Age and short-term mortality (treatment or induction-mortality) among older patients with AML-all reported relative effect (HR and RR)**


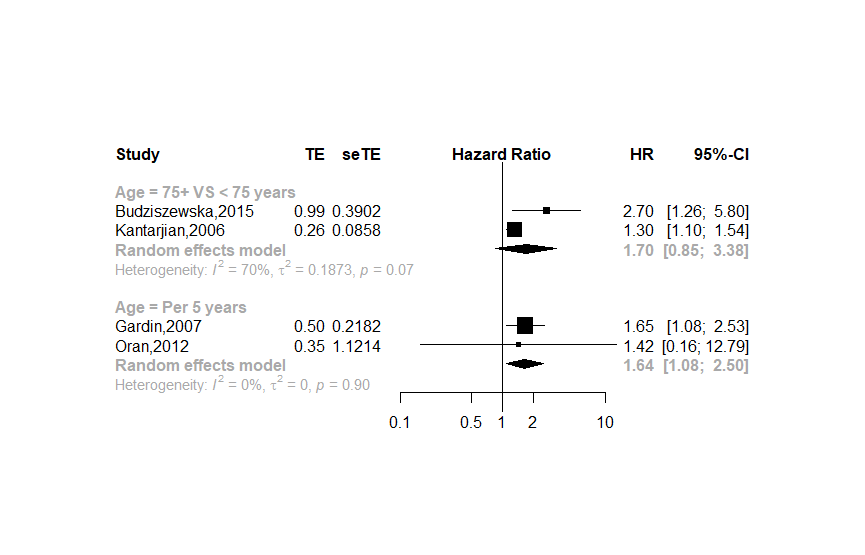

Supplement: S4 Appendix — (DOCX) [file pone.0278578.s004.docx]
