# Supplementary material for: Prognosis of older patients with newly diagnosed AML undergoing antileukemic therapy: A systematic review
Source: PLoS One. 2022 Dec 5;17(12):e0278578. doi: 10.1371/journal.pone.0278578 (PMC9721486; doi:10.1371/journal.pone.0278578)

PS and long-term mortality

Figure 1： PS and long-term mortality among older patients with AML-HR


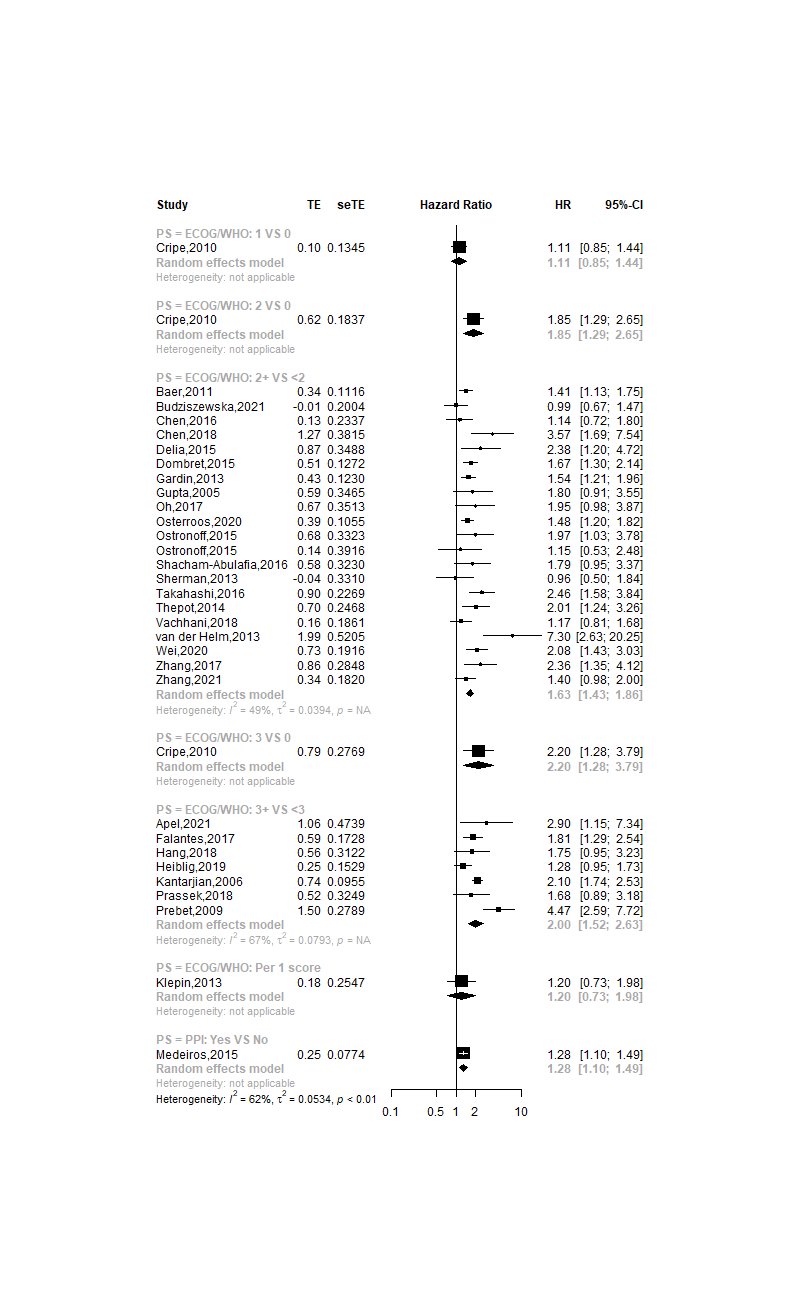


Figure 2： PS and long-term mortality among older patients with AML-RR


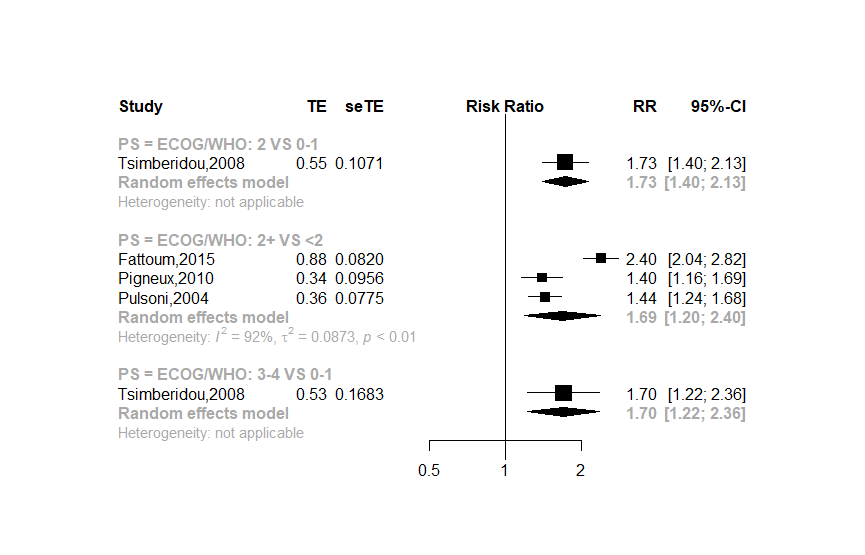


Figure 3： PS and long-term mortality among older patients with AML-OR


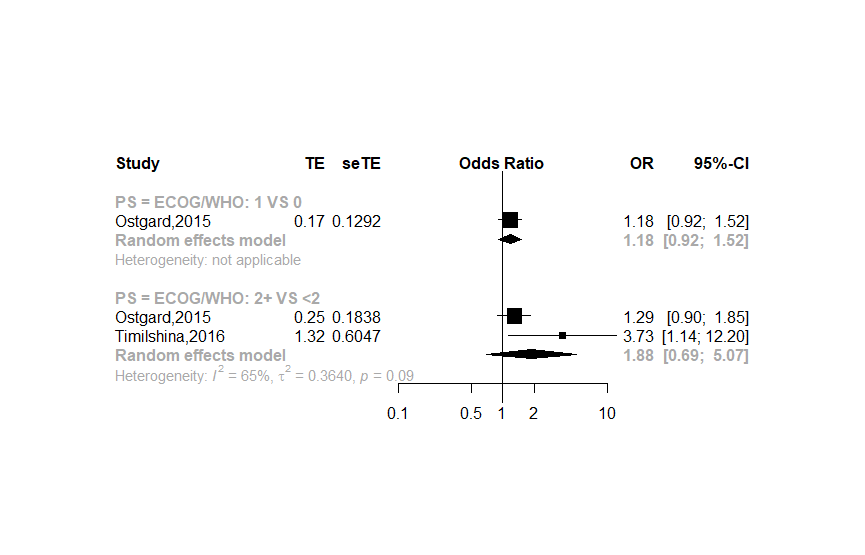


Figure 3： PS and long-term mortality among older patients with AML-OR, RR and HR


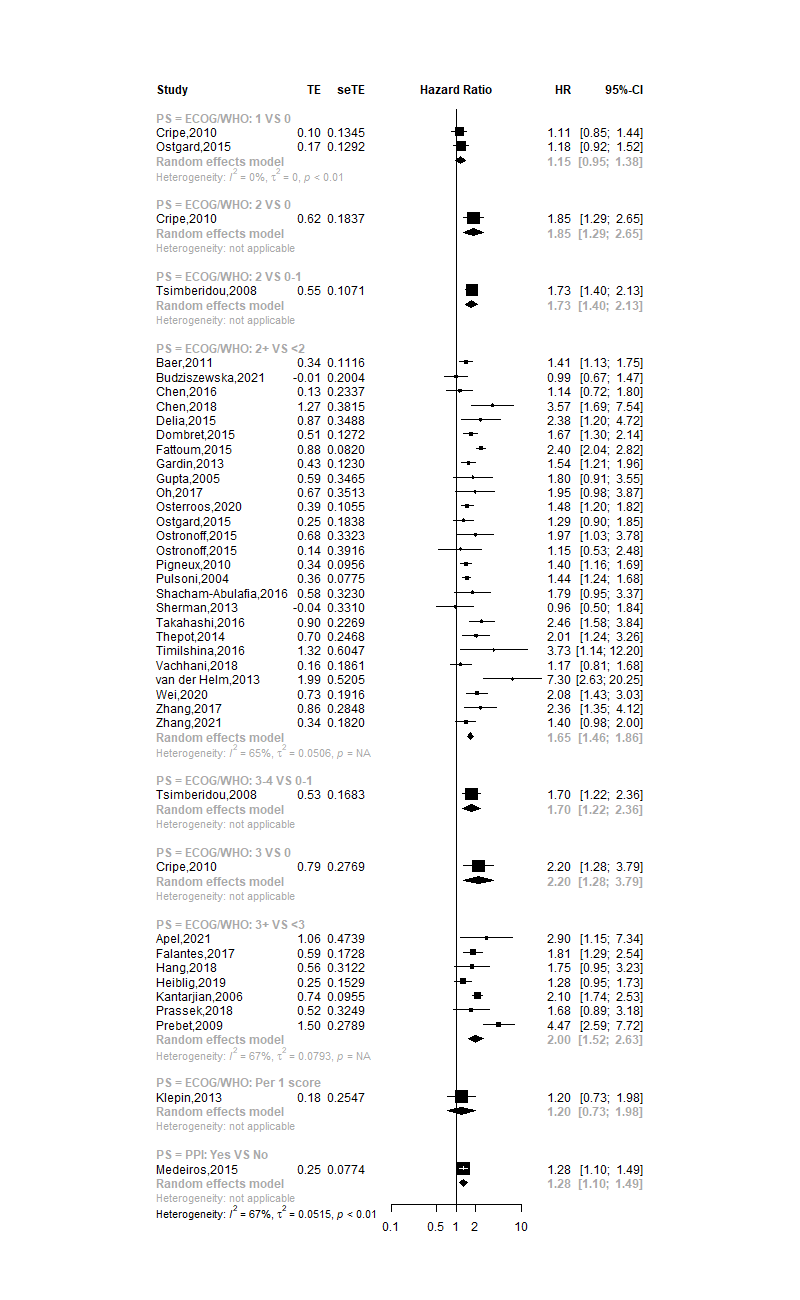


PS and short-term mortality (treatment or induction-mortality)

Figure 1: PS and short-term mortality (treatment or induction-mortality) among older patients with AML-HR


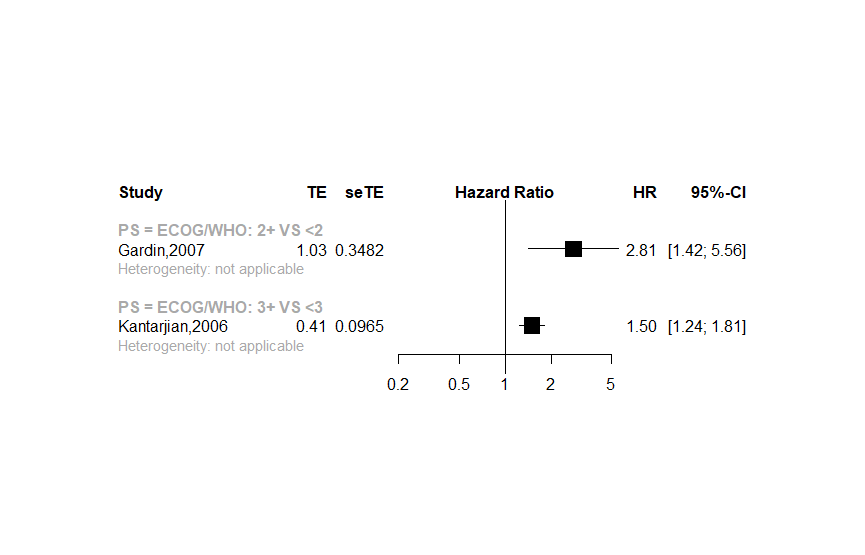


Figure 2: PS and short-term mortality (treatment or induction-mortality) among older patients with AML-OR


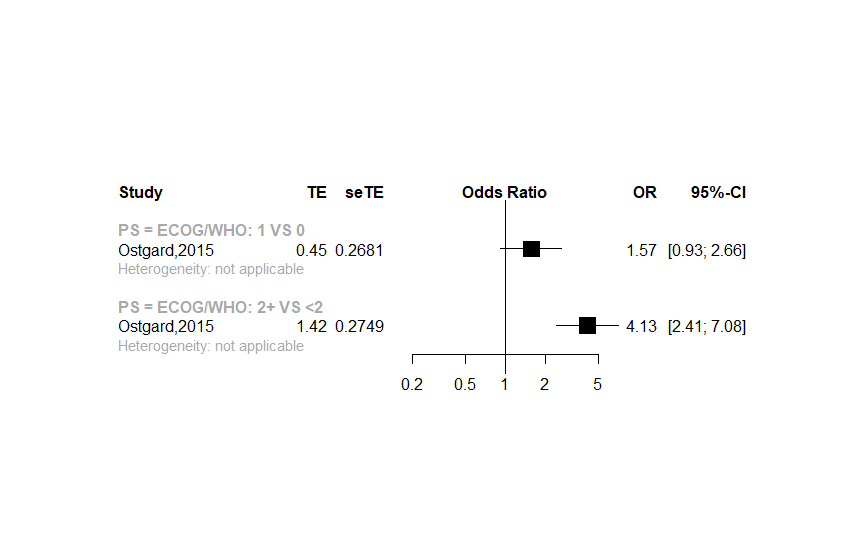


Figure 3: PS and short-term mortality (treatment or induction-mortality) among older patients with AML-All relative risk effect (HR and OR)


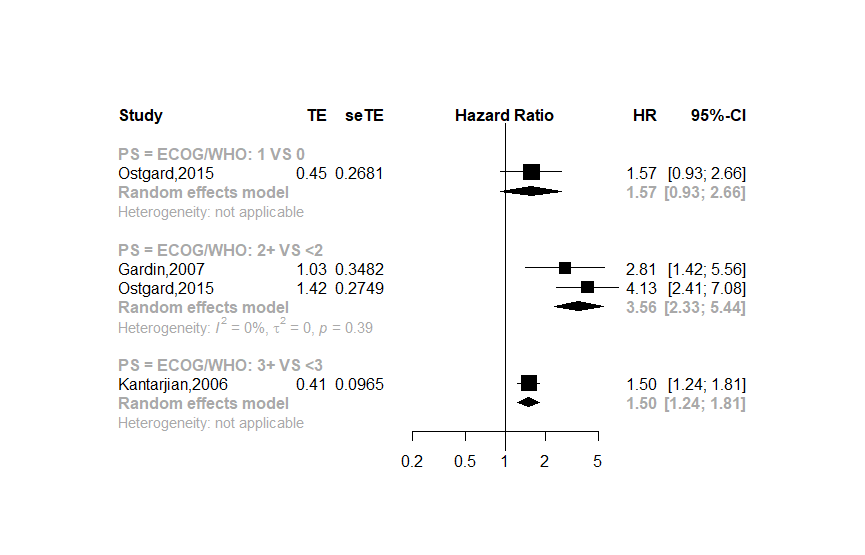

Supplement: S6 Appendix — (DOCX) [file pone.0278578.s006.docx]
