# Supplementary material for: Prognosis of older patients with newly diagnosed AML undergoing antileukemic therapy: A systematic review
Source: PLoS One. 2022 Dec 5;17(12):e0278578. doi: 10.1371/journal.pone.0278578 (PMC9721486; doi:10.1371/journal.pone.0278578)

Age (per 5 years increase) and long-term mortality

Figure 1: Subgroup analysis according to overall quality (ROB) for age assessed by per 5 years increase predicts long-term mortality among older patients with AML


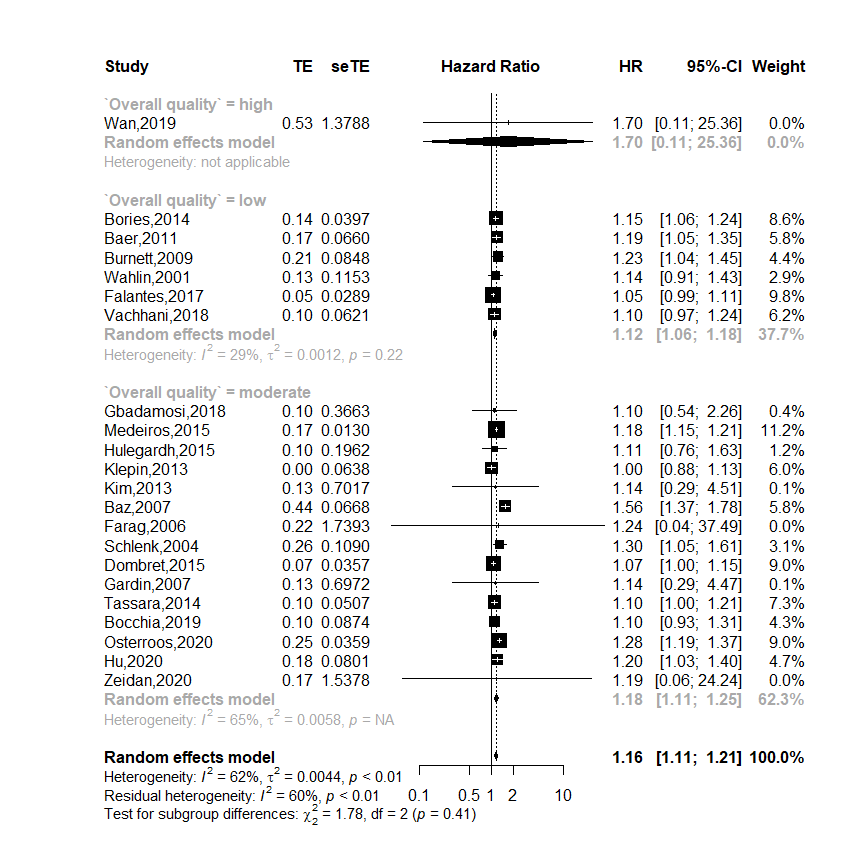


Figure 2: Subgroup analysis according to treatment type for per 5 years increase predicts long-term mortality among older patients with AML


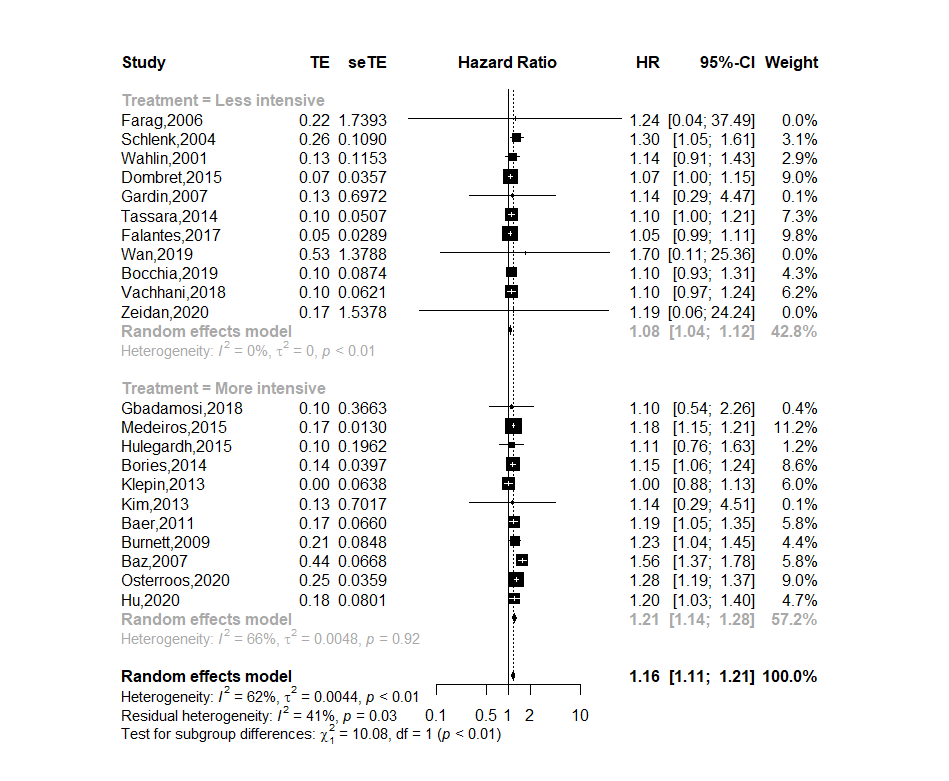


Figure 3: Subgroup analysis according to recruit time (first patient) for per 5 years increase predicts long-term mortality among older patients with AML


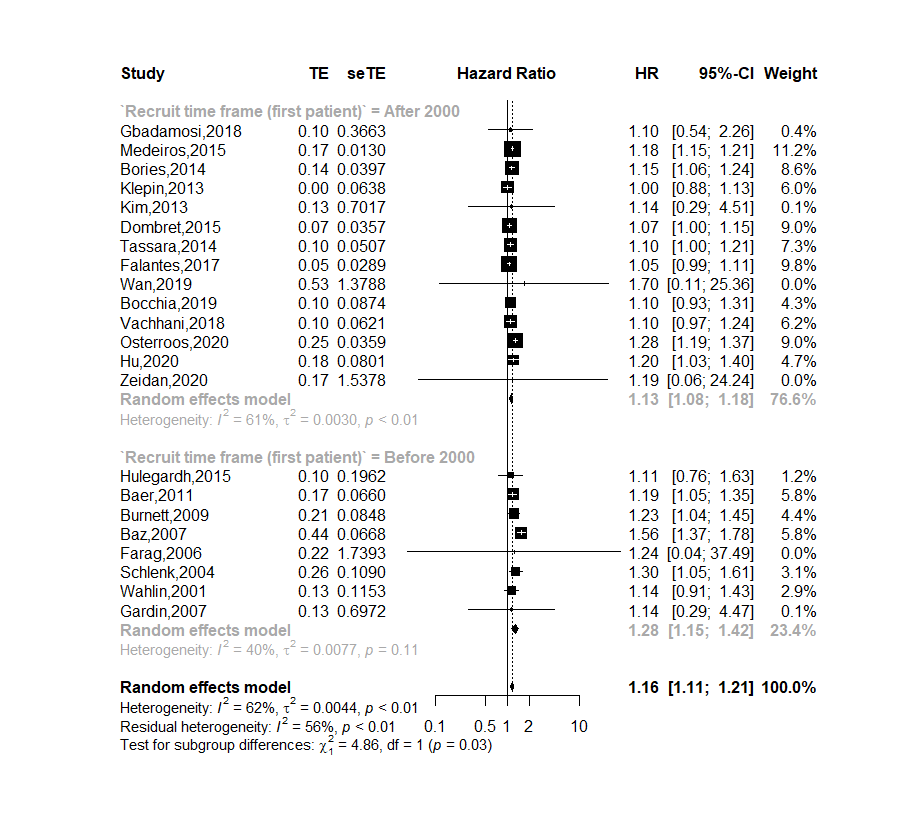


Figure 4: Funnel plot for per 5 years increase predicts long-term mortality (1+ years) among older patients with AML (Egger's test: P value= 0.99)


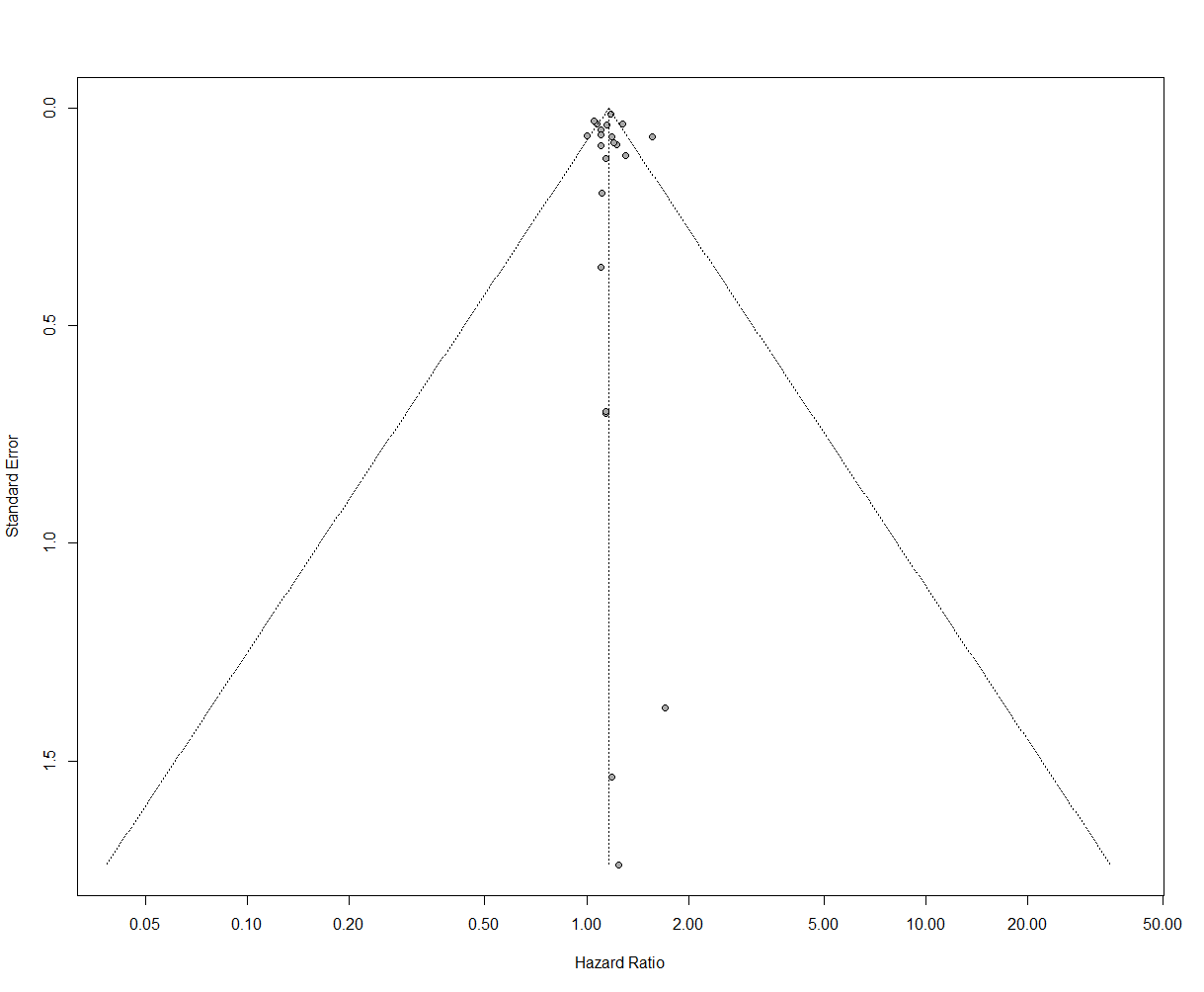


Performance score (measured by ECOG/WHO: score 2+ VS <2) and long-term mortality

Figure 1: Subgroup analysis according to overall quality (ROB) for performance assessed by ECOG/WHO (2+ VS <2) and long-term mortality among older patients with AML


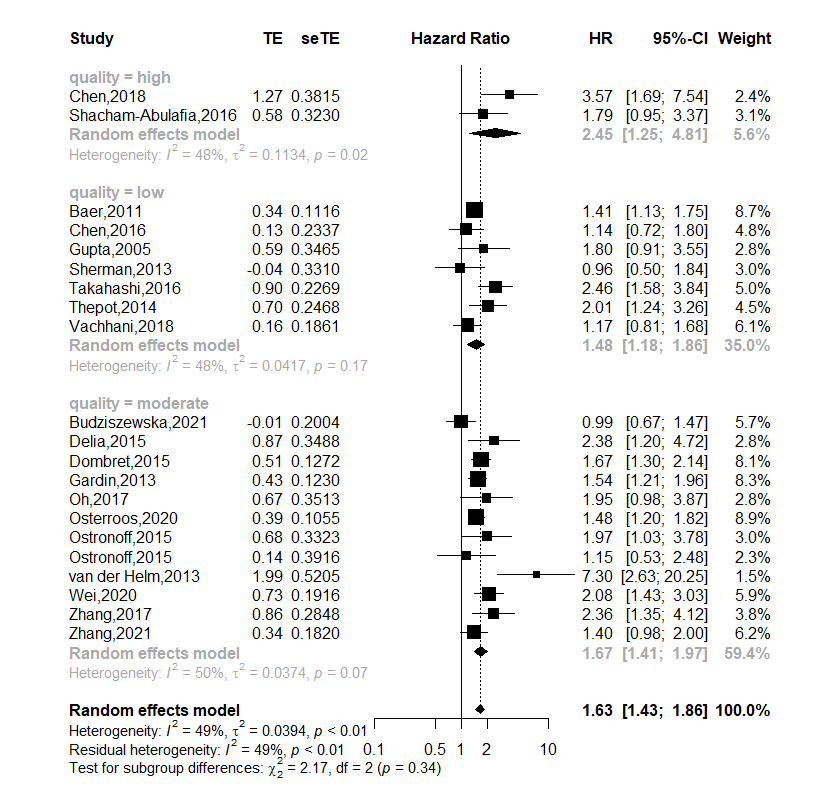


Figure 2: Subgroup analysis according to treatment type for performance assessed by ECOG/WHO (2+ VS <2) and long-term mortality among older patients with AML


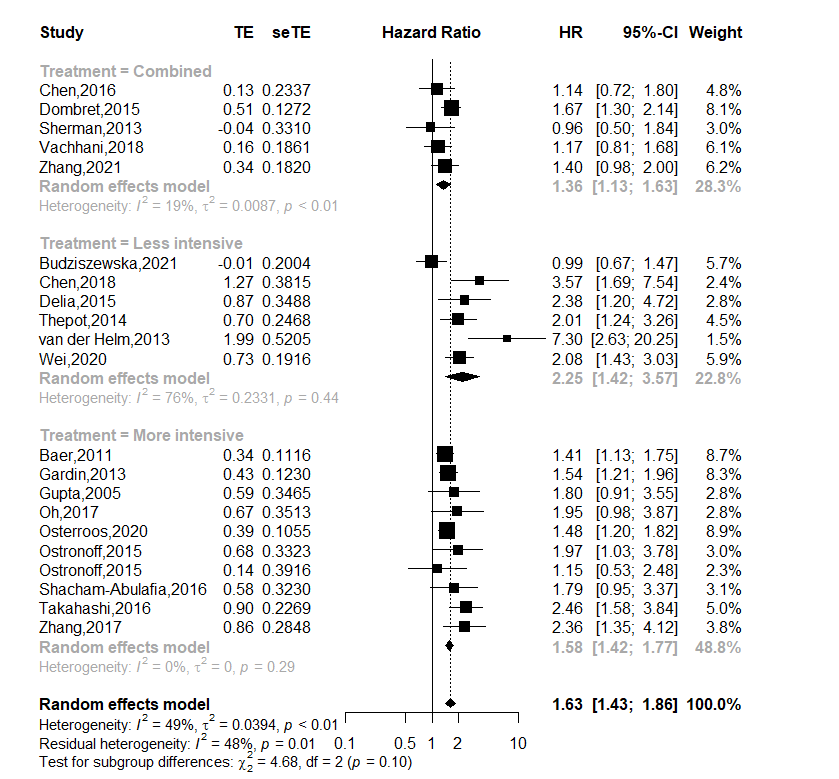


Figure 3: Subgroup analysis according to recruit time (first patient) for performance assessed by ECOG/WHO (2+ VS <2) and long-term mortality among older patients with AML


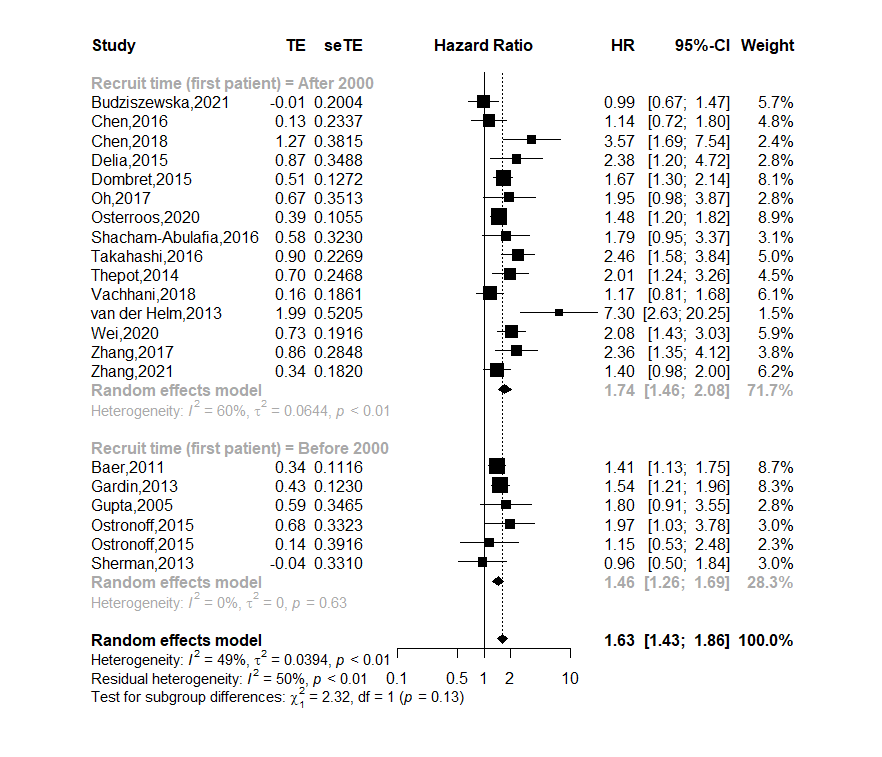


Figure 4: Funnel plot for per 5 years increase predicts long-term mortality (1+ years) among older patients with AML (Egger's test: P value= 0.07)


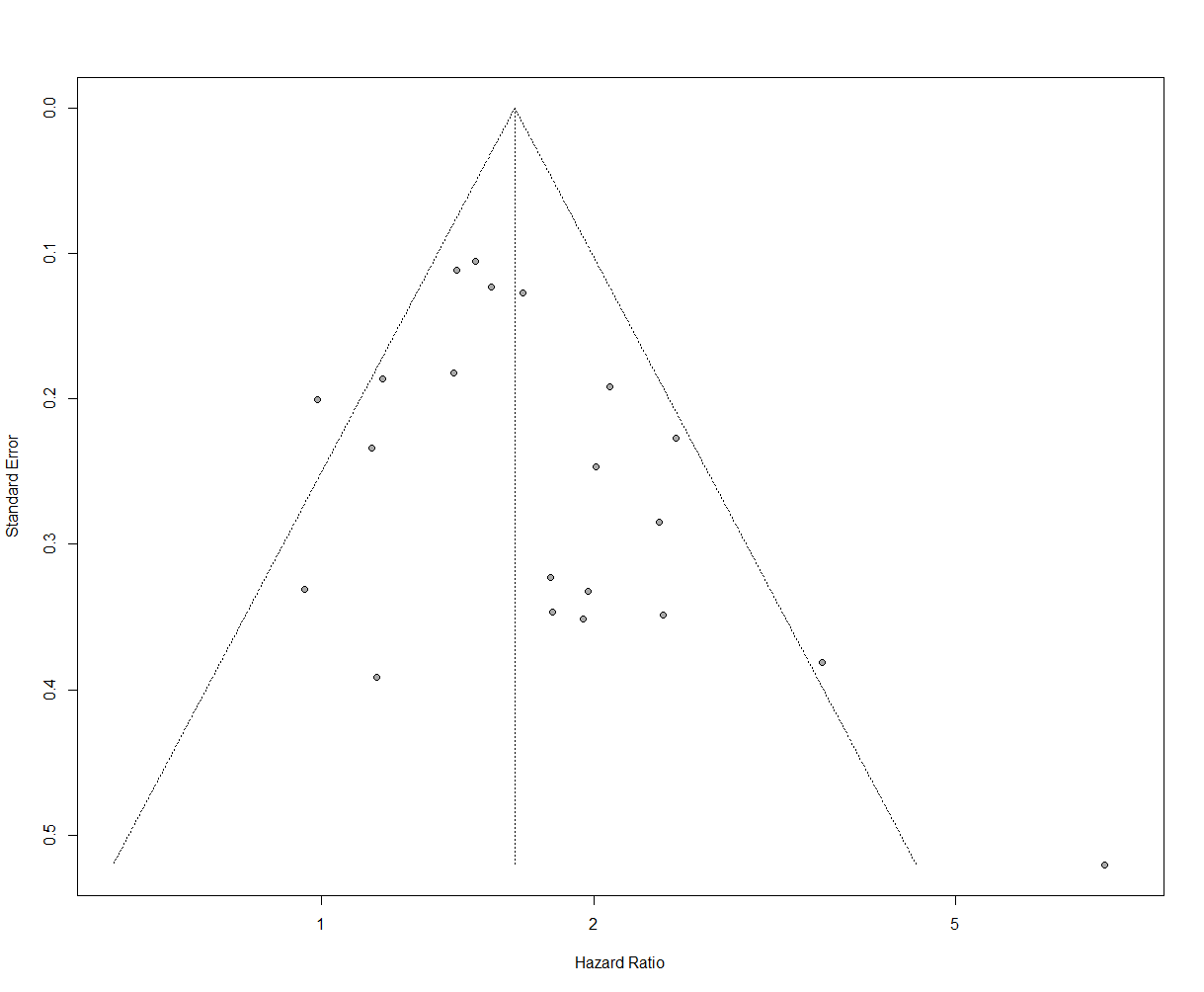

Supplement: S7 Appendix — (DOCX) [file pone.0278578.s007.docx]
