# Supplementary material for: Prognosis of older patients with newly diagnosed AML undergoing antileukemic therapy: A systematic review
Source: PLoS One. 2022 Dec 5;17(12):e0278578. doi: 10.1371/journal.pone.0278578 (PMC9721486; doi:10.1371/journal.pone.0278578)
Supplement: S1 Table — (DOCX) [file pone.0278578.s008.docx]

Table S1: Grading of recommendations, assessment, development, and evaluation—confidence in estimates of effect for short term mortality

| Number of studies (assess with HR) | Quality assessment | | | | | | Effect | Quality |
| --- | --- | --- | --- | --- | --- | --- | --- | --- |
|  | Study design | Risk of bias | Inconsistency | indirectness | Imprecision | Other considerations | HR (95% CI) |  |
| Age Per 5 years increase | | | | | | | | |
| 1 | Observational  studies | Not serious | NA | Not serious | Not serious | Very Serious* | 1.65 (1.08 to 2.53) | ⨁⨁◯◯  Low |
| Age 75 years old or more VS less than 75 years old | | | | | | | | |
| 1 | Observational  studies | Serious due to analysis and reporting issues | NA | Not serious | Not serious | Very Serious* | 1.30 (1.10 to 1.54) | ⨁◯◯◯  Very Low |
| CCI: score 1 or over VS less than 1 | | | | | | | | |
| 1 | Observational  studies | Not serious | NA | Not serious | Serious | Very Serious* | 1.60 (0.60 to 4.28) | ⨁◯◯◯  Very Low |
| HCT-CI: score 3 or over VS less than 3 | | | | | | | | |
| 1 | Observational  studies | Serious due to study confounding | NA | Not serious | Not serious | Very Serious* | 0.63 (0.46 to 0.85) | ⨁◯◯◯  Very Low |
| ECOG/WHO: score 2 or over VS less than 2 | | | | | | | | |
| 1 | Observational  studies | Not serious | NA | Not serious | Not serious | Very Serious* | 2.81 (1.42 to 5.56) | ⨁⨁◯◯  Low |
| ECOG/WHO: score 3 or over VS less than 3 | | | | | | | | |
| 1 | Observational  studies | Serious due to analysis and reporting issues | NA | Not serious | Serious | Very Serious* | 1.50 (1.24 to 1.81) | ⨁◯◯◯  Very Low |

NA: Not Applicable due to only one study reported the outcomes; * very serious due to limited data.
