# Supplementary material for: Prognosis of older patients with newly diagnosed AML undergoing antileukemic therapy: A systematic review
Source: PLoS One. 2022 Dec 5;17(12):e0278578. doi: 10.1371/journal.pone.0278578 (PMC9721486; doi:10.1371/journal.pone.0278578)
Supplement: Attachment — Submitted filename: Reviewer Report.docx [file pone.0278578.s011.docx]

Your research has been reviewed according to PRISMA 2020 Guideline.

The title is appropriate.

Introduction

The rationale for the review is described well, but it is recommended to write an introductory paragraph on quality of life and fatigue in AML and it can be changed with the paragraph between lines 77-83.

Objectives are clearly defined.

“We conducted a … of older patients with AML” should be stated in the objectives.

Methods

Inclusion and exclusion criteria for the review are specified clearly, but you should also state how studies were grouped for the syntheses in this paragraph.

Information sources, search strategy, selection process and data collection, data items, methods used to access the risk of bias, effect measures, synthesis methods, bias assessment, and certainty assessment are specified.

Results

Study selection is described, and studies are cited.

Characteristics of studies are presented.

The risk of bias in studies is presented.

Results of the individual studies and results of syntheses are given appropriately.

For each synthesis, the risk of bias is presented.

Certainty of evidence is presented.

Discussion

The discussion provides a general interpretation of the results and discusses the limitations of the paper.
