# Supplementary material for: Prognosis of older patients with newly diagnosed AML undergoing antileukemic therapy: A systematic review
Source: PLoS One. 2022 Dec 5;17(12):e0278578. doi: 10.1371/journal.pone.0278578 (PMC9721486; doi:10.1371/journal.pone.0278578)
Supplement: Attachment — Submitted filename: Response to Reviewers.docx [file pone.0278578.s012.docx]

Ahmet Emre Eşkazan, M.D.

Academic Editor

PLOS ONE

Dear Dr. Ahmet Emre Eşkazan,

We have received and reviewed the editors' and reviewers’ comments and instructions on our manuscript. Thank you very much for the opportunity to revise our manuscript. A point-by-point response to the comments follows. We highlighted all the changes made to the original manuscript. We (the authors) received no specific funding for this work and have declared that no competing interests exist. We also provided the abstracted outcome data set (minimal data set) for our analysis.

My colleagues and I are grateful for your consideration of our work, and we look forward to hearing your decision.

Qiukui Hao and Romina Brignardello-Petersen, for the review team

**Journal Requirements:**

When submitting your revision, we need you to address these additional requirements.

1. Please ensure that your manuscript meets PLOS ONE's style requirements, including those for file naming. The PLOS ONE style templates can be found at

https://journals.plos.org/plosone/s/file?id=wjVg/PLOSOne_formatting_sample_main_body.pdf and

https://journals.plos.org/plosone/s/file?id=ba62/PLOSOne_formatting_sample_title_authors_affiliations.pdf

**Reply:** We changed the format accordingly.

2. Thank you for stating the following financial disclosure: "NA"

At this time, please address the following queries:

a) Please clarify the sources of funding (financial or material support) for your study. List the grants or organizations that supported your study, including funding received from your institution.

b) State what role the funders took in the study. If the funders had no role in your study, please state: “The funders had no role in study design, data collection and analysis, decision to publish, or preparation of the manuscript.”

c) If any authors received a salary from any of your funders, please state which authors and which funders.

d) If you did not receive any funding for this study, please state: “The authors received no specific funding for this work.”

Please include your amended statements within your cover letter; we will change the online submission form on your behalf.

**Reply:** We added the statement accordingly.

3. Thank you for stating the following in your Competing Interests section: "None"

Please complete your Competing Interests on the online submission form to state any Competing Interests. If you have no competing interests, please state "The authors have declared that no competing interests exist.", as detailed online in our guide for authors at http://journals.plos.org/plosone/s/submit-now

This information should be included in your cover letter; we will change the online submission form on your behalf.

**Reply:** We added the statement accordingly.

4. In your Data Availability statement, you have not specified where the minimal data set underlying the results described in your manuscript can be found. PLOS defines a study's minimal data set as the underlying data used to reach the conclusions drawn in the manuscript and any additional data required to replicate the reported study findings in their entirety. All PLOS journals require that the minimal data set be made fully available. For more information about our data policy, please see http://journals.plos.org/plosone/s/data-availability.

Upon re-submitting your revised manuscript, please upload your study’s minimal underlying data set as either Supporting Information files or to a stable, public repository and include the relevant URLs, DOIs, or accession numbers within your revised cover letter. For a list of acceptable repositories, please see http://journals.plos.org/plosone/s/data-availability#loc-recommended-repositories. Any potentially identifying patient information must be fully anonymized.

Important: If there are ethical or legal restrictions to sharing your data publicly, please explain these restrictions in detail. Please see our guidelines for more information on what we consider unacceptable restrictions to publicly sharing data: http://journals.plos.org/plosone/s/data-availability#loc-unacceptable-data-access-restrictions. Note that it is not acceptable for the authors to be the sole named individuals responsible for ensuring data access.

We will update your Data Availability statement to reflect the information you provide in your cover letter.

**Reply:** We provided the abstracted outcome data set for our analysis in appendix S10.

5. Please include captions for your Supporting Information files at the end of your manuscript, and update any in-text citations to match accordingly. Please see our Supporting Information guidelines for more information: http://journals.plos.org/plosone/s/supporting-information.

**Reply:** We added this section accordingly.

Reviewers' comments:

Reviewer's Responses to Questions

Reviewer #1: This is a good effort to perform a meta analysis to define contributing factors to long term mortality in older adults with AML. While the results are no surprise and not substantially different than the data available to treating physicians, the effort to pull in published data and assess quality of evidence is a good effort.

1. Authors need to clarify how they reconciled the subgroup effect of treatment and treatment time period on age effect on mortality. It may be critical as with new non intensive but effective treatments may blunt some of the effects of age on mortality

**Reply: The reviewer raises an interesting point that we have now addressed in the Discussion as follows:**

*We found that the impact of age on increasing mortality was marginally greater in those receiving more intensive therapy (HR 1,21) than in those receiving less intensive therapy (HR 1.21) and was also marginally greater in studies in which the first patient was enrolled before 2000 (HR 1,28) than those recruited after 2000 (HR 1.13). These findings suggest that the increased risk of dying with older age may depend on what chemotherapy regimen one is receiving. It thus further suggests that as new therapies are developed the association of age with mortality may change. In particular, new non-intensive but effect treatments may blunt some of the association between age and mortality.*

2. The authors have made minimal reference to not using cytogenetics as their contention is favorable or intermediate risk AML does not exist in older population. THis can be generally true but again with recent advances in effective therapies show that cytogenetics or disease based risk categories do matter. Authors need to highlight this potential shortcoming in discussion

**Reply:** we added related information in our discussion section. The sentence reads:

*“**However, some cytogenetic abnormalities may provide prognostic value as some new emerging personalized treatments may target some gene mutations or biomarkers ^1^”*

3. Statement in line 389 needs to be modified "Clinicians using the recommendations can use our results to identify which older patients are considered appropriate for antileukemic therapy". I would rather use the results presented to choose therapy or encourage participation in clinical trials so that we can improve outcomes in this high risk population and not to discourage therapy.

**Reply:** we revised the sentence accordingly. The sentence reads:

*“**Clinicians can use the tools and thresholds for which we found high and moderate quality evidence (e.g., age: 70 or 75 years, HCT-CI: score 3, ECOG/WHO: score 2) to do pre-antileukemic therapy assessment and engage in shared decision-making with patients (e.g., choose therapy or encourage participation in clinical trials) to improve outcomes in this population. Furthermore, clinicians or researchers should consider that QoL or fatigue are important outcomes for patients…”*

4. Authors claim that their analysis links QoL with age, comorbidities but the number of studies are limited. This needs to be acknowledged

**Reply:** we acknowledged this limitation in evidence in our revised manuscript.

*“**The limited evidence available on QOL and lack of evidence on frailty and functional status restrict our understanding of the prognostic values of factors on these outcomes.”*

Reviewer #2: Your research has been reviewed according to PRISMA 2020 Guideline.

The title is appropriate.

Introduction

The rationale for the review is described well, but it is recommended to write an introductory paragraph on quality of life and fatigue in AML and it can be changed with the paragraph between lines 77-83.

**Reply:** We added some description about quality of life and fatigue in older AML patients. The sentence reads:

*“In patients with AML, fatigue is one of the common symptoms and is related to quality of life^2^. Even though older patients with AML may have similar quality of life (QOL) between intensive and non-intensive chemotherapy^3^, QOL is an important outcome to consider for treatment decisions ^4^and clinical trials^5^.*

Objectives are clearly defined.

“We conducted a … of older patients with AML” should be stated in the objectives.

**Reply:** we revised this sentence accordingly. The sentence reads:

*“Therefore, we conducted a systematic review to determine the association between these prognostic factors and mortality and health-related quality of life or fatigue of older patients with newly diagnosed acute myeloid leukemia (AML) undergoing antileukemic therapy.”*

Methods

Inclusion and exclusion criteria for the review are specified clearly, but you should also state how studies were grouped for the syntheses in this paragraph.

**Reply:** we added the information accordingly. The sentence reads:

*“We group all eligible studies for syntheses according to the reported outcomes and the cut-off values of the above prognostic factors.”*

Information sources, search strategy, selection process and data collection, data items, methods used to access the risk of bias, effect measures, synthesis methods, bias assessment, and certainty assessment are specified.

Results

Study selection is described, and studies are cited.

Characteristics of studies are presented.

The risk of bias in studies is presented.

Results of the individual studies and results of syntheses are given appropriately.

For each synthesis, the risk of bias is presented.

Certainty of evidence is presented.

Discussion

The discussion provides a general interpretation of the results and discusses the limitations of the paper.

**Reply:** Many thanks for the positive comments about our manuscript.

While revising your submission, please upload your figure files to the Preflight Analysis and Conversion Engine (PACE) digital diagnostic tool, https://pacev2.apexcovantage.com/. PACE helps ensure that figures meet PLOS requirements. To use PACE, you must first register as a user. Registration is free. Then, login and navigate to the UPLOAD tab, where you will find detailed instructions on how to use the tool. If you encounter any issues or have any questions when using PACE, please email PLOS at figures@plos.org. Please note that Supporting Information files do not need this step.

**Reply:** We convert our figure accordingly.

**New references**

1. Pourrajab F, Zare-Khormizi MR, Hashemi AS, et al. Genetic Characterization and Risk Stratification of Acute Myeloid Leukemia. *Cancer Manag Res* 2020;12:2231-53. doi: 10.2147/cmar.S242479 [published Online First: 2020/04/11]

2. Alibhai SMH, Breunis H, Matelski J, et al. Age-related cytokine effects on cancer-related fatigue and quality of life in acute myeloid leukemia. *J Geriatr Oncol* 2020;11(3):402-09. doi: 10.1016/j.jgo.2019.04.009 [published Online First: 2019/05/14]

3. El-Jawahri A, Abel GA, Traeger L, et al. Quality of life and mood of older patients with acute myeloid leukemia (AML) receiving intensive and non-intensive chemotherapy. *Leukemia* 2019;33(10):2393-402. doi: 10.1038/s41375-019-0449-1 [published Online First: 2019/03/30]

4. Oliva EN, Nobile F, Alimena G, et al. Quality of life in elderly patients with acute myeloid leukemia: patients may be more accurate than physicians. *Haematologica* 2011;96(5):696-702. doi: 10.3324/haematol.2010.036715 [published Online First: 2011/02/19]

5. Roboz GJ, Döhner H, Pocock C, et al. Oral azacitidine preserves favorable level of fatigue and health-related quality of life for patients with acute myeloid leukemia in remission: results from the phase 3, placebo-controlled QUAZAR AML-001 trial. *Haematologica* 2021;106(12):3240-44. doi: 10.3324/haematol.2021.279174 [published Online First: 2021/09/24]
